# Native QRS duration and outcomes in heart failure with mildly reduced ejection fraction: results from a large-scaled registry

**DOI:** 10.1007/s00392-025-02667-8

**Published:** 2025-05-12

**Authors:** Finn Ole Kronberg, Michael Behnes, Marielen Reinhardt, Noah Abel, Alexander Schmitt, Felix Lau, Thomas Bertsch, Henning Johann Steffen, Kathrin Weidner, Mohammad Abumayyaleh, Jürgen Kuschyk, Ibrahim Akin, Tobias Schupp

**Affiliations:** 1 Department of Cardiology, Angiology, Haemostaseology and Medical Intensive Care, University Medical Centre Mannheim, Medical Faculty Mannheim, Heidelberg University, Mannheim, Germany; 2 Institute of Clinical Chemistry, Laboratory Medicine and Transfusion Medicine, Nuremberg General Hospital, Paracelsus Medical University, Nuremberg, Germany

**Keywords:** Heart failure with mildly reduced ejection fraction, HFmrEF, QRS, QRS duration

## Abstract

**Objective:**

The study investigates the prognostic impact of the native QRS duration in patients with heart failure and mildly reduced ejection fraction (HFmrEF).

**Background:**

The prognostic impact of QRS duration in HFmrEF has rarely been investigated.

**Methods:**

Consecutive patients with HFmrEF and available 12-lead electrocardiogram were retrospectively included at one institution from 2016 to 2022. Patients with QRS duration ≥ 120 ms were compared to patients with QRS duration < 120 ms, further risk stratification was performed comparing patients with left and right bundle branch block (LBBB vs. RBBB). The primary endpoint was all-cause mortality at 30 months, secondary endpoints comprised the risk of HF-related rehospitalization.

**Results:**

In total, 1627 patients with HFmrEF were included with a median QRS duration of 90 ms (i.e., QRS duration ≥ 120 ms: 15%). Although the risk of long-term all-cause mortality was not affected by a prolonged QRS duration (35.1% vs. 28.7%; p = 0.057; HR = 1.254; 95% CI 0.993–1.583), patients with QRS duration ≥ 120 ms had a higher risk of HF-related rehospitalization (18.2% vs. 11.9%; p = 0.008; HR = 1.574; 95% CI 1.124–2.204). A QRS duration ≥ 120 ms was associated with long-term HF-related rehospitalization even after multivariable adjustment (HR 1.420, 95% CI 1.008–2.002, p = 0.045). Finally, the risks of long-term all-cause mortality and HF-related rehospitalization did not differ among patients with LBBB and RBBB.

**Conclusion:**

A prolonged native QRS duration is independently associated with a higher risk of HF-related rehospitalization in HFmrEF, but not long-term all-cause mortality.

**Graphical abstract:**

**Figure:**
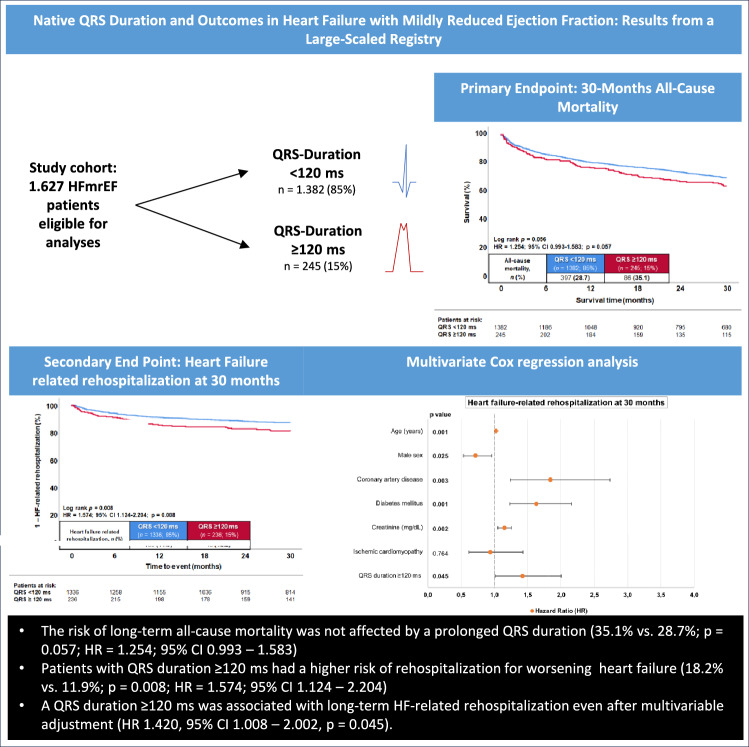

## Introduction

Heart failure (HF) is a complex clinical syndrome with an increasing lifetime risk related to demographic changes. By now, HF affects up to 4% of the general population [1, 2] with a corresponding 1-year mortality rate of 8% and tremendous 5 year mortality rate after HF-related hospitalization of up to 75% [3, 4]. Besides the established HF phenotypes (i.e., HF with reduced and preserved ejection fraction (i.e., HFrEF and HFpEF), data from the ESC HF Long-Term Registry suggested one fourth of the patients hospitalized with HF are diagnosed with HF with mildly reduced ejection fraction (i.e., HFmrEF; left ventricular ejection fraction (LVEF) 41–49%). Recently, the risk of 5-year mortality was shown to be comparable among the three HF phenotypes [3, 4]. Similarities between patients with HFmrEF and HFrEF include a younger age and higher rates of male sex and coronary artery disease (CAD), whereas patients with HFmrEF were older and suffered from higher rates of concomitant atrial fibrillation, which represent typical patterns in HFpEF [3, 5].

In patients with HFrEF, a prolonged QRS duration is a recognized predictor of adverse long-term prognosis, due to interventricular delay and a subsequent reduction of left ventricular output [6–8]. From this perspective, cardiac resynchronization therapy (CRT) was yet demonstrated to improve LVEF and reduce both the risks of all-cause mortality and HF-related hospitalization in patients with HFrEF [9, 10]. Since HFmrEF was recently demonstrated to be closely linked to HFrEF, including the response to HF pharmacotherapies, it may be hypothesized that the QRS duration may affect outcomes in patients with HFmrEF.

Therefore, the present study sought to investigate the prognostic impact of native QRS duration on long-term outcomes of patients hospitalized with HFmrEF.

## Methods

### Study patients, design and data collection

For the present study, all consecutive patients hospitalized with HFmrEF at one University Medical Centre were included from January 2016 to December 2022 [11]. Using the electronic hospital information system, all relevant clinical data related to the index event were documented, such as baseline characteristics, vital signs on admission, prior medical history, prior medical treatment, length of index hospital and intensive care unit (ICU) stay, laboratory values, data derived from all non-invasive or invasive cardiac diagnostics and device therapies, such as echocardiographic data, coronary angiography, data being derived from prior or newly implanted cardiac devices and HF-related pharmacotherapies at index hospital discharge. Every re-visit at the outpatient clinic or rehospitalizations related to HF or adverse cardiac events were documented until the end of the year 2022.

The present study derived from the “Heart Failure With Mildly Reduced Ejection Fraction Registry” (HARMER), representing a retrospective single-center registry including consecutive patients with HFmrEF hospitalized at the University Medical Center Mannheim (UMM), Germany (clinicaltrials.gov identifier: NCT05603390). The registry was carried out according to the principles of the declaration of Helsinki and was approved by the medical ethics committee II of the Medical Faculty Mannheim, University of Heidelberg, Germany (ethical approval code: 2022–818).

### Inclusion and exclusion criteria

All consecutive patients with ≥ 18 years of age hospitalized with HFmrEF at one institution were included. The diagnosis of HFmrEF was determined according to the “2021 ESC Guidelines for the diagnosis and treatment of acute and chronic heart failure” [12]. Accordingly, all patients with LVEF 41–49% and symptoms and/or signs of HF were included. The presence of elevated amino-terminal prohormone of brain natriuretic peptide (NT-proBNP) levels and other evidence of structural heart disease were considered to make the diagnosis more likely, but were not mandatory for diagnosis of HFmrEF. Transthoracic echocardiography was performed by cardiologists during routine clinical care being blinded to the final study analysis in accordance with current European guidelines [13]. For the present sub-study, patients without documented 12-lead electrocardiogram (ECG) during index hospitalization were excluded. Furthermore, patients with an implanted pacemaker (prior or during index hospitalization) or implanted CRT were excluded.

### Risk stratification

Firstly, patients with a native QRS duration ≥ 120 ms were compared to patients with a native QRS duration < 120 ms. Thereafter, the prognosis of patients with RBBB was compared to LBBB. RBBB and LBBB were defined in accordance with the AHA/ACC/HRS recommendations. Accordingly, RBBB was defined as the combination of a QRS duration ≥ 120 ms, an rsr′, rsR′, or rSR′ in leads V_1_or V_2_ and an S wave in I or V_6_ > 40 ms or of greater duration than R wave. LBBB was defined as a QRS duration ≥ 120 ms, with a broad notched or slurred R wave in leads I, aVL, V_5_, and V_6_ and an occasional RS pattern in V_5_ and V_6_ with absent q waves in leads I, V_5_, and V_6_, R peak time greater than 60 ms in leads V_5_ and V_6_ and ST and T waves usually opposite in direction to QRS [14]. For the present study, all 12-lead ECG data was reassessed post-hoc by an independent cardiologist.

### Study endpoints

The primary endpoint was all-cause mortality during a median follow-up of 30 months. Secondary endpoints comprised in-hospital all-cause mortality, all-cause mortality at 12 months, rehospitalization for worsening HF, cardiac rehospitalization, acute myocardial infarction (AMI), stroke, coronary revascularization and major adverse cardiac and cerebrovascular events (MACCE) during follow-up. From a total of 2228 patients with HFmrEF, 44 patients with no evidence on long-term follow-up were excluded (corresponding lost-to follow-up rate: 1.97%). HF-related hospitalization was defined as a rehospitalization due to worsening HF requiring intravenous diuretic therapy. HF-related rehospitalization comprised patients with hospitalization due to worsening HF as the primary cause or as a result of another cause but associated with worsening HF at the time of admission, or as a result of another cause but complicated by worsening HF during its cause. Cardiac rehospitalization was defined as rehospitalization due to a primary cardiac condition, including worsening HF, AMI, coronary revascularization and symptomatic atrial or ventricular arrhythmias. MACCE was defined as composite of all-cause mortality, coronary revascularization, non-fatal AMI and non-fatal stroke.

### Statistical methods

Quantitative data is presented as mean ± standard error of mean (SEM), median and interquartile range (IQR), and ranges depending on the distribution of the data. They were compared using the Student’s t test for normally distributed data or the Mann–Whitney *U* test for nonparametric data. Deviations from a Gaussian distribution were tested by the Kolmogorov–Smirnov test. Qualitative data is presented as absolute and relative frequencies and were compared using the Chi-square test or the Fisher’s exact test, as appropriate. Kaplan–Meier analyses were performed comparing patients with and without prolonged QRS duration, respectively for patients with RBBB and LBBB. Univariable hazard ratios (HR) were given together with 95% confidence intervals. The prognostic impact of QRS duration was thereafter investigated within multivariable Cox regression models. For Cox regression models first, we performed univariable Cox regression analyses including patients’ characteristics and comorbidities that were yet demonstrated to affect outcomes in HF patients. Only parameters with p ≤ 0.10 within univariable Cox regression models were included into multivariable Cox regression analyses. Results of all statistical tests were considered significant for p ≤ 0.05. SPSS (Version 28, IBM, Armonk, New York) was used for statistics.

## Results

### Study population

**Fig. 1:**
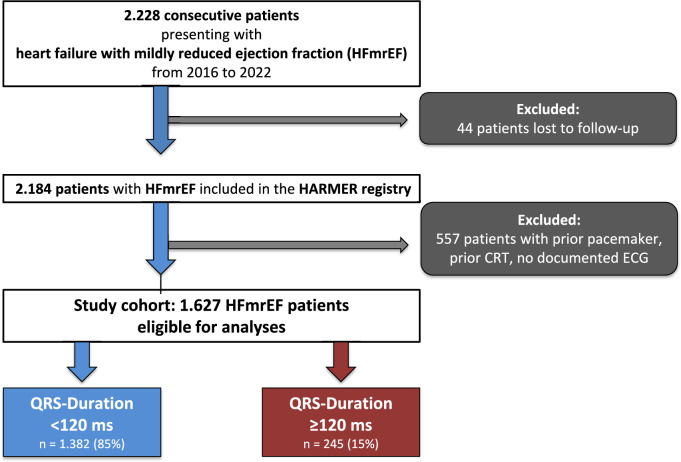
Study flow chart

From 2016 to 2022, 2228 patients with HFmrEF were hospitalized at our institution. Of those, 44 patients with incomplete follow-up and 283 patients with an implanted pacemaker or CRT (prior or during index hospitalization), as well as 274 patients without documented ECG during index hospitalization were excluded. The final study cohort comprised a total of 1,627 patients with HFmrEF (Fig. 1). Within the entire study cohort, the median native QRS duration was 90 ms (IQR 80–100 ms), whereas 245 patients (15%) had a prolonged QRS duration of ≥ 120 ms. Patients with a QRS duration ≥ 120 ms were older (median age 80 years vs. 73 years, p = 0.001) and more often males (71.8% vs. 64.5%, p = 0.027). The rates of prior coronary artery disease (CAD) (46.1% vs. 39.4%, p = 0.047), prior coronary artery bypass graft (CABG) (14.3% vs. 8.7%, p = 0.006) and valve surgery (6.5% vs. 2.8%, p = 0.003) were higher in patients with prolonged QRS duration, alongside with higher rates of prior congestive HF (40.8% vs. 30.0%, p = 0.001) (Table 1). Ischemic cardiomyopathy (ICMP) was the most common HF etiology in both groups, whereas the distribution of HF etiologies did not differ among patients with and without prolonged QRS duration (62.0% vs. 59.8%, p = 0.408) (Table 2). Patients with a QRS duration ≥ 120 ms had a higher NYHA functional class (NYHA functional class III + IV: 34.3% vs. 26.6%; p = 0.001) in line with higher rates of moderate-severe aortic stenosis (13.9% vs. 9.0%, p = 0.017) and mitral regurgitation (17.6% vs. 11.3%, p = 0.006). With regard to laboratory data on admission, patients with a prolonged QRS duration had a lower estimated glomerular filtration rate (eGFR) (median 59 mL/min/1.73m^2^ vs. 68 mL/min/1.73m^2^, p = 0.001) and lower white blood cell count (median 7.85 × 10^9^/L vs. 8.37 × 10^9^/L, p = 0.006). Further biomarkers, such as haemoglobin, NT-proBNP, cardiac troponin I and C-reactive protein (CRP) did not differ between both groups.

**Table 1:** Baseline characteristics

	QRS < 120 ms (*n* = 1382)		QRS ≥ 120 ms (*n* = 245)		p value
Age, median (IQR)	73	(62–81)	80	(72–85)	**0.001**
Male sex, n (%)	892	(64.5)	176	(71.8)	**0.027**
Body mass index, kg/m^2^, median (IQR)	26.8	(23.8–30.9)	26.5	(24.0–30.5)	0.790
SBP, mmHg, median (IQR)	143	(125–161)	140	(122–160)	0.438
DBP, mmHg, median (IQR)	80	(70–92)	74	(63–87)	**0.001**
Heart rate, bpm, median (IQR)	83	(70–99)	76	(65–89)	**0.001**
Medical history, n (%)					
Coronary artery disease	544	(39.4)	113	(46.1)	**0.047**
Prior myocardial infarction	337	(24.4)	63	(25.7)	0.656
Prior PCI	393	(28.4)	77	(31.4)	0.341
Prior CABG	120	(8.7)	35	(14.3)	**0.006**
Prior valvular surgery	39	(2.8)	16	(6.5)	**0.003**
Congestive heart failure	415	(30.0)	100	(40.8)	**0.001**
Decompensated heart failure < 12 months	137	(9.9)	37	(15.1)	**0.015**
Prior ICD	23	(1.7)	6	(2.4)	0.392
Prior sICD	7	(0.5)	0	(0.0)	0.264
Chronic kidney disease	380	(27.5)	85	(34.7)	**0.022**
Peripheral artery disease	155	(11.2)	36	(14.7)	0.119
Stroke	194	(14.0)	37	(15.1)	0.660
Liver cirrhosis	18	(1.3)	7	(2.9)	0.068
Malignancy	218	(15.8)	36	(14.7)	0.668
Cardiovascular risk factors, n (%)					
Arterial hypertension	1063	(76.9)	201	(82.0)	0.076
Diabetes mellitus	485	(35.1)	93	(38.0)	0.388
Hyperlipidaemia	403	(29.2)	86	(35.1)	0.062
Smoking					
Current	282	(20.4)	31	(12.7)	**0.005**
Former	248	(17.9)	51	(20.8)	0.285
Family history	145	(10.5)	23	(9.4)	0.601
Comorbidities at index hospitalization, n (%)					
Acute coronary syndrome					
Unstable angina	68	(4.9)	15	(6.1)	0.431
STEMI	149	(10.8)	11	(4.5)	**0.002**
NSTEMI	195	(14.1)	35	(14.3)	0.942
Acute decompensated heart failure	309	(22.4)	66	(26.9)	0.117
Cardiogenic shock	33	(2.4)	10	(4.1)	0.128
Atrial fibrillation	553	(40.0)	103	(42.0)	0.551
Cardiopulmonary resuscitation	36	(2.6)	8	(3.3)	0.557
Out-of-hospital	15	(1.1)	3	(1.2)	0.848
In-hospital	21	(1.5)	5	(2.0)	0.549
Stroke	160	(11.6)	31	(12.7)	0.630
COPD	164	(11.9)	29	(11.8)	0.989
Medication on admission, n (%)					
ACE-inhibitor	480	(34.7)	92	(37.6)	0.394
ARB	309	(22.4)	59	(24.1)	0.552
Beta-blocker	755	(54.6)	141	(57.6)	0.397
Aldosterone antagonist	110	(8.0)	22	(9.0)	0.590
ARNI	11	(0.8)	1	(0.4)	0.513
SGLT2-inhibitor	33	(2.4)	2	(0.8)	0.118
Loop diuretics	469	(33.9)	106	(43.3)	**0.005**
Statin	590	(42.7)	123	(50.2)	**0.029**
ASA	472	(34.2)	91	(37.1)	0.365
P2Y12-inhibitor	148	(10.7)	21	(8.6)	0.312
DOAC	312	(22.6)	65	(26.5)	0.176
Vitamin K antagonist	92	(6.7)	13	(5.3)	0.428

*ACE* angiotensin-converting-enzyme, *ARB* angiotensin receptor blocker, *ARNI* angiotensin receptor neprilysin inhibitor, *ASA* acetylsalicylic acid, *CABG* coronary artery bypass grafting, *CKD* chronic kidney disease, *COPD* chronic obstructive pulmonary disease, *CRT-D* cardiac resynchronization therapy with defibrillator, *DBP* diastolic blood pressure, *DOAC* directly acting oral anticoagulant, *IQR* interquartile range, *LTOT* long-term oxygen therapy, *(N)STEMI* non-ST-segment elevation myocardial infarction, SBP systolic blood pressure, *SGLT2* sodium glucose linked transporter 2, *(s) ICD* (subcutaneous) implantable cardioverter defibrillator

Level of significance p ≤ 0.05. Bold type indicates statistical significance

**Table 2:** Heart-failure related and procedural data

	QRS < 120 ms (*n* = 1382)		QRS ≥ 120 ms (*n* = 245)		p value
Heart failure etiology, n (%)					
Ischemic cardiomyopathy	827	(59.8)	152	(62.0)	0.408
Non-ischemic cardiomyopathy	88	(6.4)	14	(5.7)	
Hypertensive cardiomyopathy	104	(7.5)	22	(9.0)	
Congenital heart disease	1	(0.1)	0	(0.0)	
Valvular heart disease	53	(3.8)	10	(4.1)	
Tachycardia-associated	67	(4.8)	5	(2.0)	
Tachymyopathy	32	(2.3)	2	(0.8)	
Unknown	210	(15.2)	40	(16.3)	
NYHA functional class, n (%)					
I/II	1014	(73.4)	161	(65.7)	**0.001**
III	242	(17.5)	67	(27.3)	
IV	126	(9.1)	17	(6.9)	
Electrocardiographic data					
Heart rate, bpm, median (IQR)	78 (66–93)		73 (63–86)		**0.001**
PQ-interval, ms, median (IQR)	160 (140–180)		170 (150–200)		**0.001**
QRS-interval, ms, median (IQR)	90 (80–100)		140 (130–150)		**0.001**
QT-interval, ms, median (IQR)	360 (340–400)		400 (375–440)		**0.001**
Echocardiographic data					
LVEF, %, median (IQR)	45 (45–47)		45 (45–47)		0.434
IVSd, mm, median (IQR)	12 (10–13)		12 (11–13)		**0.001**
LVEDD, mm, median (IQR)	49 (44–54)		50 (45–54)		0.156
TAPSE, mm, median (IQR)	20 (18–23)		21 (18–24)		**0.040**
LA diameter, mm, median (IQR)	41 (36–47)		42 (38–49)		**0.048**
LA surface, cm^2^, median (IQR)	21.0 (17.0–25.0)		22.0 (17.0–26.0)		0.312
E/A, median (IQR)	0.8 (0.7–1.2)		0.7 (0.6–1.1)		0.075
E/E`, median (IQR)	9.0 (6.3–13.5)		10.0 (6.0–15.2)		0.453
Diastolic dysfunction, n (%)	989	(71.6)	185	(75.5)	0.204
Moderate-severe aortic stenosis, n (%)	124	(9.0)	34	(13.9)	**0.017**
Moderate-severe aortic regurgitation, n (%)	57	(4.1)	8	(3.3)	0.527
Moderate-severe mitral regurgitation, n (%)	156	(11.3)	43	(17.6)	**0.006**
Moderate-severe tricuspid regurgitation, n (%)	190	(13.7)	42	(17.1)	0.161
VCI, mm, median (IQR)	19 (15–25)		21 (17–25)		0.257
Aortic root, mm, median (IQR)	33 (30–36)		33 (30–37)		0.571
Coronary angiography, n (%)	657	(47.5)	110	(44.9)	0.445
No evidence of coronary artery disease	124	(18.9)	22	(20.0)	0.057
1-vessel disease	121	(18.4)	19	(17.3)	
2-vessel disease	153	(23.3)	14	(12.7)	
3-vessel disease	259	(39.4)	55	(50.0)	
CABG	46	(7.0)	17	(15.5)	**0.003**
Chronic total occlusion	87	(13.2)	14	(12.7)	0.883
PCI, n (%)	368	(56.0)	48	(43.6)	**0.016**
Sent to CABG, n (%)	37	(5.6)	4	(3.6)	0.389
Baseline laboratory values, median (IQR)					
Potassium, mmol/L	3.9 (3.6–4.2)		3.9 (3.6–4.2)		0.869
Sodium, mmol/L	139 (137–141)		139 (137–142)		0.421
Creatinine, mg/dL	1.04 (0.85–1.37)		1.19 (0.88–1.54)		**0.001**
eGFR, mL/min/1.73 m^2^	68 (48–88)		59 (43–77)		**0.001**
Haemoglobin, g/dL	12.5 (10.5–14)		12.0 (10.3–13.6)		0.085
WBC count, × 10^9^/L	8.37 (6.56–10.32)		7.85 (6.25–9.42)		**0.006**
Platelet count, × 10^9^/L	228 (180–289)		224 (176–275)		0.250
HbA1c, %	5.9 (5.5–6.7)		6.1 (5.5–7.1)		0.172
LDL- cholesterol, mg/dL	99 (76–128)		91 (71–128)		0.259
HDL- cholesterol, mgl/dL	42 (34–51)		42 (35–50)		0.956
C-reactive protein, mg/L	14 (3–46)		13 (4–48)		0.990
NT-pro BNP, pg/mL	2607 (947–6828)		2607 (1140–5777)		0.125
Cardiac troponin I, µg/L	0.03 (0.02–0.29)		0.03 (0.02–0.12)		0.334
Medication at discharge, n (%)					
ACE-inhibitor	710	(53.1)	118	(50.0)	0.373
ARB	307	(23.0)	65	(27.5)	0.128
Beta-blocker	1070	(80.1)	177	(75.0)	0.075
Aldosterone antagonist	172	(12.9)	40	(16.9)	0.091
ARNI	15	(1.1)	1	(0.4)	0.324
SGLT2-inhibitor	60	(4.5)	12	(5.1)	0.688
Loop diuretics	613	(45.9)	124	(52.5)	0.059
Statin	926	(69.3)	170	(72.0)	0.401
Digitalis	58	(4.3)	7	(3.0)	0.328
Amiodarone	37	(2.8)	4	(1.7)	0.340
ASA	717	(53.7)	122	(51.7)	0.575
P2Y12-inhibitor	491	(36.8)	71	(30.1)	**0.049**
DOAC	448	(33.5)	83	(35.2)	0.624
Vitamin K antagonist	70	(5.2)	12	(5.1)	0.921
LMWH	89	(6.7)	11	(4.7)	0.246

*ACE* angiotensin-converting enzyme, *ARB* angiotensin receptor blocker, *ARNI* angiotensin receptor neprilysin inhibitor, *ASA* acetylsalicylic acid, *CABG* coronary artery bypass grafting, *DOAC* directly acting oral anticoagulant, *DPP4* dipeptidyl-peptidase 4, *eGFR* estimated glomerular filtration rate, *GLP1* glucagon like peptide, *HbA1c* glycated haemoglobin, *HDL* high-density lipoprotein, *IQR* interquartile range, *IVSd* Interventricular septal end diastole, *LA* left atrial, *LDL* low-density lipoprotein, *LMWH* low molecular weight heparin, *LVEDD* Left ventricular end-diastolic diameter, *LVEF* left ventricular ejection fraction, *NT-pro BNP* aminoterminal pro-B-type natriuretic peptide, *NYHA* New York Heart Association, *PCI* percutaneous coronary intervention, *SGLT2* sodium glucose linked transporter 2, *TAPSE* tricuspid annular plane systolic excursion, *VCI* Vena cava inferior, *WBC* white blood cells

Level of significance p ≤ 0.05. Bold type indicates statistical significance

### Correlations of QRS duration with echocardiographic, ECG and laboratory data

In patients with HFmrEF, the QRS duration correlated with age (*r* = 0.139; p = 0.001), interventricular septum diameter (IVSd) (*r* = 0.141; p = 0.001), left ventricular end-diastole diameter (LVEDD) (*r* = 0.161; p = 0.001), tricuspid annular plane systolic excursion (TAPSE) (*r* = 0.050; p = 0.042) as well as PQ (*r* = 0.188; p = 0.001) and QT intervals (*r* = 0.374; p = 0.001), whereas negative correlations with estimated glomerular filtration rate (eGFR) (*r* = – 0.081; p = 0.001), white blood cell (WBC) (*r* = -0.078; p = 0.002) and platelet counts (*r* = – 0.055; p = 0.027) were observed (Table 3).

**Table 3:** Correlations of QRS interval with echocardiographic, laboratory and clinical parameters

	QRS duration (ms)	
	r	p value
Age (years)	0.139	**0.001**
Body mass index (kg/m^2^)	0.043	0.104
LVEF (%)	– 0.029	0.240
IVSd (mm)	0.141	**0.001**
LVEDD (mm)	0.161	**0.001**
TAPSE (mm)	0.050	**0.042**
eGFR (ml/min1.73m^2^)	– 0.081	**0.001**
Haemoglobin (g/dl)	– 0.011	0.656
WBC count (× 10^9^/L)	– 0.078	**0.002**
Platelet count (× 10^9^/L)	– 0.055	**0.027**
C-reactive protein (mg/L)	– 0.031	0.242
NT-proBNP (pg/mL)	– 0.012	0.766
PQ interval (ms)	0.188	**0.001**
QT interval (ms)	0.374	**0.001**

*eGFR* estimated glomerular filtration rate, *IVSd* Interventricular septal end diastole, *LVEDD* left ventricular end-diastolic diameter, *LVEF* left ventricular ejection fraction, *NT-pro BNP* aminoterminal pro-B-type natriuretic peptide, *TAPSE* tricuspid annular plane systolic excursion, *WBC* white blood cell count

Level of significance p ≤ 0.05. Bold type indicates statistical significance

### Prognostic impact of QRS duration in patients with HFmrEF

**Fig. 2:**
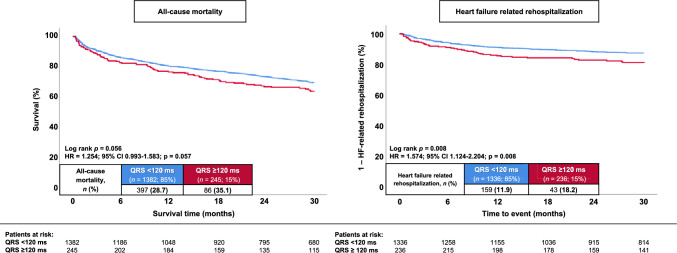
Kaplan–Meier analyses investigating the prognostic impact of the QRS duration in patients with HFmrEF with regard to the risk of all-cause mortality (left panel) and HF-related rehospitalization (right panel)

During a median follow-up of 30 months, the risk of all-cause mortality was not significantly affected by the QRS duration (≥ 120 ms vs. < 120 ms: 35.1% vs. 28.7%; p = 0.057; HR = 1.254; 95% CI 0.993–1.583). Regarding key secondary endpoints, patients with a QRS duration ≥ 120 ms had a higher risk of HF-related rehospitalization at 30 months (18.2% vs. 11.9%; p = 0.008; HR = 1.574; 95% CI 1.124–2.204) (Table 4**; **Fig. 2).

**Table 4:** Follow-up data, primary and secondary endpoints

	QRS < 120 ms (*n* = 1382)		QRS ≥ 120 ms (*n* = 245)		HR	95% CI	p value
Primary endpoint, n (%)							
All-cause mortality, at 30 months	397	(28.7)	86	(35.1)	1.254	0.993–1.583	0.057
Secondary endpoints, n (%)							
All-cause mortality, in-hospital	46	(3.3)	9	(3.7)	1.108	0.535–2.293	0.783
All-cause mortality, at 12 months	275	(19.9)	58	(23.7)	1.221	0.920–1.621	0.167
Heart-failure related rehospitalization, at 30 months	159	(11.9)	43	(18.2)	1.574	1.124–2.204	**0.008**
Cardiac rehospitalization, at 30 months	297	(22.2)	67	(28.4)	1.299	0.997–1.694	0.053
Coronary revascularization, at 30 months	108	(8.1)	18	(7.6)	0.926	0.562–1.525	0.763
Acute myocardial infarction, at 30 months	45	(3.4)	7	(3.0)	0.858	0.387–1.902	0.706
Stroke, at 30 months	32	(2.4)	6	(2.5)	1.048	0.438–2.506	0.916
MACCE, at 30 months	514	(37.2)	106	(43.3)	1.186	0.963–1.462	0.109
Follow-up data, median (IQR)							
Hospitalization time, days	8 (5–15)		10 (6–15)		–	–	0.221
ICU time, days	0 (0–1)		0 (0–1)		–	–	0.625
Follow-up time, days	884 (378–1556)		857 (362–1467)		–	–	0.263

*ADHF* acute decompensated heart failure, *CI* confidence interval, *HF* heart failure, *HR* hazard ratio, *ICU* intensive care unit, *IQR* interquartile range, *MACCE* major adverse cardiac and cerebrovascular events, *TAPSE* tricuspid annular plane systolic excursion

Level of significance p ≤ 0.05. Bold type indicates statistical significance

Further secondary endpoints, such as all-cause mortality during hospitalization, at 12-months, as well as cardiac rehospitalization, coronary revascularization, AMI, stroke or MACCE, each at 30 months, did not differ in patients with or without prolonged QRS duration.

**Fig. 3:**
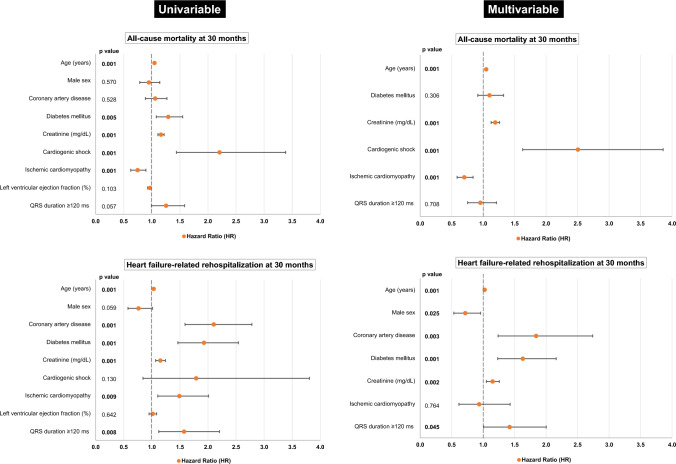
Forrest plots illustrating uni- (left panel) and multivariable (right panel) Cox regression analyses with regard to the risk of all-cause mortality and HF-related rehospitalization

After multivariable adjustment, QRS duration ≥ 120 ms was not associated with the risk of long-term all-cause mortality (HR 0.956, 95% CI 0.754–1.211, p = 0.708). However, advanced age (HR 1.049, 95% CI 1.040–1.058, p = 0.001; per year increase), elevated creatinine levels (HR 1.192, 95% CI 1.127–1.260, p = 0.001; per 1 mg/dl increase) and the presence of cardiogenic shock during index hospitalization (HR 2.505, 95% CI 1.627–3.857, p = 0.001) were associated with an increased risk of long-term all-cause mortality. However, a QRS duration ≥ 120 ms was an independent risk factor for HF-related rehospitalization at 30 months after multivariable adjustment (HR 1.420, 95% CI 1.008–2.002, p = 0.045) (Fig. 3).

### Prognostic impact of LBBB vs. RBBB

**Fig. 4:**
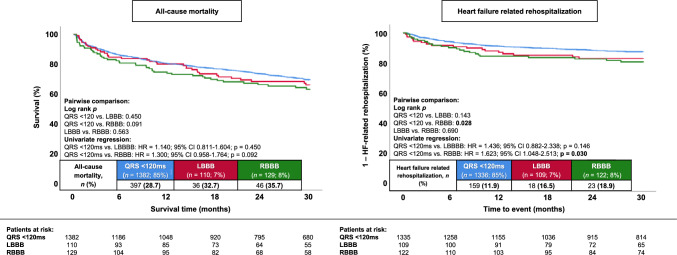
Kaplan–Meier analyses investigating the prognosis of patients with LBBB, RBBB and QRS duration < 120 ms and HFmrEF with regard to the risk of all-cause mortality (left panel) and HF-related rehospitalization (right panel)

To further evaluate the impact of the type of bundle branch block, patients with prolonged QRS duration were stratified by the presence of left LBBB (n = 110) or RBBB (n = 129). A total of 6 patients had a prolonged QRS duration ≥ 120 ms with neither LBBB nor RBBB and were excluded in the following sub-analysis. Both, the risks of all-cause mortality at 30 months (32.7% in the LBBB cohort vs. 35.7% in the RBBB cohort, p = 0.563; HR 1.137, 95% CI 0.735–1.759) and HF-related rehospitalization at 30 months (16.5% in LBBB cohort vs. 18.9% in RBBB cohort, p = 0.690; HR 1.134, 95% CI 0.612–1.101) did not significantly differ between the two groups (Fig. 4). However, specifically the presence of RBBB was associated with a higher risk of HF-related rehospitalization compared to patients with QRS duration < 120 ms (18.9% vs. 11.9%; p = 0.030; HR 1.623, 95% CI 1.048–2.513).

## Discussion

The aim of the present study was to investigate the prognostic impact of a prolonged native QRS duration in consecutive patients hospitalized with HFmrEF. A prolonged QRS duration ≥ 120 ms was observed in 15% of patients with HFmrEF and was more common in older patients with increased burden of cardiac comorbidities. However, a QRS duration ≥ 120 ms was not associated with the risk of long-term all-cause mortality, but an increased risk of HF-related rehospitalization, even after multivariable adjustment. Increased risk of HF-related rehospitalization in patients with prolonged QRS was specifically seen in patients with RBBB, whereas the prognosis of patients with HFmrEF and a LBBB or a RBBB did not significantly differ.

By now, various predictors for a prolonged QRS duration have yet been identified, predominantly in patients with HFrEF. For instance, male sex and older age were associated with a prolonged QRS duration, which may relate to different proportions of the heart and thorax between the sexes and decreasing conduction speed with rising age [15–17]. This is in line with the present study, suggesting a positive correlation of age with the QRS duration, which is of major importance given ongoing demographic changes and the aging population.

The higher prevalence of CAD, prior CABG and valvular surgery in the prolonged QRS duration cohort suggests a widening of the QRS duration through ischemic or traumatic injury of the cardiac conduction system [18]. Furthermore, the prevalence of LBBB in the HFmrEF collective was higher compared to ours within the ESC Heart Failure Long-Term Registry (15.4% vs 6.7%). An explanation for this might be the lower rates of ICMP and higher rates of idiopathic dilated cardiomyopathies (IDCM) (28%) in the respective study, which are associated with higher rates of LBBB compared to ICMP [3, 19]. In a meta-analysis of the DAPA-HF and DELIVER collectives 28% of HFmrEF collective had a QRS duration > 120 ms. 9% of their whole collective had a conventional pacemaker which could explain the difference to our prevalence of 15% BBB here [20]. Despite the rather low prevalence of a prolonged native QRS duration, a prolonged QRS duration ≥ 120 ms was independently associated with a 1.4 fold increased risk of HF-related rehospitalization in HFmrEF. 

The prognostic impact of native QRS duration was yet investigated in various clinical settings including patients with AMI [21–24], HFrEF [25–27] or HFpEF [28, 29]. QRS prolongation is more common in HFrEF being present in about 30% of patients and associated with an impaired outcome [19, 30, 31]. In line, a QRS duration ≥ 120 ms was associated with the progression of HF after 1 year follow up in 153 patients in NYHA I with LVEF < 50%, while most of those patients had HFrEF (i.e., 52%) [32]. In a database analysis of the Swedish HF and the CHECK-HF registries with 8852 eligible patients with HFmrEF, a wide QRS duration (≥ 130 ms) seemed to be a risk factor for all-cause mortality and HF hospitalization, although patients with device-induced widening of the QRS complex as well as patients with native wide QRS complexes were included in this analysis. However, a major part of the study population (i.e., 85.7%) had an implanted cardiac device, predominantly pacemakers (69.1%), whereas no further risk stratification was performed according to the presence of native versus device-induced prolonged QRS duration [33]. The present study confirms the negative prognostic impact of prolonged native QRS duration with regard to the risk of HF-related rehospitalization, but not long-term all-cause mortality. The cardiac rehospitalization rates were barely not significant between the normal and the prolonged QRS duration group. These findings suggest that a prolonged QRS specifically increases the risk of HF-related rehospitalization. In patients with HFmrEF, prolonged QRS duration may lead to more HF-related rehospitalizations by desynchronized ventricular contraction, ventricular dilatation and subsequent functional mitral regurgitation [34]. These mechanisms may interrupt the hemodynamic function of the heart, resulting in fluid overload and decompensated HF.

With decreasing LVEF, the incidence of BBB increases [35]. The interventricular delay leads to a reduced cardiac output. By pacing biventricular, amongst others the QRS narrows and the LVEF rise [36]. This effect is best seen in patients with a LVEF between 26 and 35% and may reduce both the risks of all-cause mortality and HF-related rehospitalization [10]. Most of those studies focused on patients with HFrEF and included patients with LVEF ≤ 35% or even ≤ 30% (RAFT and MADIT-CRT respectively) and only few included patients with higher LVEF (i.e., REVERSE (LVEF ≤ 40%) or BLOCK-HF (≤ 50%) trials) [37–39]. However, even in the BLOCK-HF trial, no further subgroup analysis was performed to estimate the benefit on patients with HFmrEF. Therefore, the present study may provide important insights regarding the prognostic impact of a prolonged native QRS duration in all-comers patients with HFmrEF, suggesting specifically an increased risk of HF-related rehospitalization at 30 months.

With rising LVEF through CRT, prognosis improves significantly even with permanent wide QRS complex with CRT therapy, suggesting QRS length impact on prognosis is weaker with device therapy [40, 41]. Reverse remodeling effects are discussed as the reason, reducing the incidence of ventricular tachyarrhythmia with rising LVEF [42–44]. High responders to CRT have wider QRS durations at baseline, indicating QRS duration has a high impact on CRT efficacy [44, 45]. From this perspective, it may be questionable, whether supply with CRT may improve outcomes in patients with HFmrEF. Although HFmrEF is characterized by “intermediate” characteristics compared to patients with HFrEF and HFpEF, recent studies suggested HFmrEF may be more linked to HFrEF than HFpEF. For instance, in the CHARM registry as well as in the SwedeHF registry, patients’ characteristics such as age, sex distribution, history of AMI and ischemic heart disease were more similar in HFrEF and HFmrEF than in HFpEF [46, 47]. This may underlines a similar etiology and pathophysiology of HFmrEF, being a minor form of HFrEF, which was also observed when investigating the effect of HF pharmacotherapies [48].

Finally, the presence of LBBB or RBBB did not influence the prognosis of patients with HFmrEF in the present study, whereas specifically RBBB was associated with an increased risk of HF-related rehospitalization as compared to patients with normal QRS duration. Direct comparisons of the prognostic impact of LBBB compared to RBBB are limited. Within a recent study, RBBB was a high-risk marker for mortality in patients with cardiogenic shock and accompanied by higher risk of 30-day all-cause mortality compared to LBBB [50]. However, studies of HFrEF and RBBB showed no beneficial outcome of CRT therapy, although the conduction delay should be reduced through biventricular pacing [51]. The pathophysiological mechanisms of electrical and mechanical dyssynchrony and response to CRT treatment remain unclear and further studies need to investigate these. Therefore, the prognostic impact of CRT in patients with prolonged QRS duration in HFmrEF needs to be investigated within further prospective studies.

## Study limitations

The retrospective and single-centre study design need to be acknowledged as a major limitation. The proportion of patients with prolonged QRS duration was rather small and may not have been large enough to demonstrate significant differences regarding the primary endpoint. Furthermore, recurrent hospitalizations were only recorded at our institution. For the present study, risk stratification was performed according to a single ECG and no ECG data was recorded during follow-up. There was no correction made for multiple testing and there is a chance that some results from these analyses may be false positive.

Finally, the causes of death beyond index hospitalization were beyond the scope of the study.

## Conclusions

The present study suggests that a prolonged native QRS duration above 120 ms represents an independent risk factor for HF-related rehospitalization, but not for all-cause mortality at 30 months in consecutive patients with HFmrEF.

## Data Availability

The datasets used and/or analyzed during the current study are available from the corresponding author upon reasonable request.
