# Correction: Native QRS duration and outcomes in heart failure with mildly reduced ejection fraction: results from a large-scaled registry

**DOI:** 10.1007/s00392-025-02683-8

**Published:** 2025-06-02

**Authors:** Finn Ole Kronberg, Michael Behnes, Marielen Reinhardt, Noah Abel, Alexander Schmitt, Felix Lau, Thomas Bertsch, Henning Johann Steffen, Kathrin Weidner, Mohammad Abumayyaleh, Jürgen Kuschyk, Ibrahim Akin, Tobias Schupp

**Affiliations:** 1 Department of Cardiology, Angiology, Haemostaseology and Medical Intensive Care, University Medical Centre Mannheim, Medical Faculty Mannheim, Heidelberg University, Mannheim, Germany; 2 Institute of Clinical Chemistry, Laboratory Medicine and Transfusion Medicine, Nuremberg General Hospital, Paracelsus Medical University, Nuremberg, Germany

**Correction: Clinical Research in Cardiology** 10.1007/s00392-025-02667-8

When this article was published, the Declaration of Ethical Approval ended like this: " … and all patients gave their informed consent prior to their inclusion in the study."

though it should correctly read:

"No written informed consent was necessary for the study."

The Original Article has been corrected.

